# Selection of medicines in Chilean public hospitals: an exploratory study

**DOI:** 10.1186/1472-6963-13-10

**Published:** 2013-01-07

**Authors:** Juan F Collao, Felicity Smith, Nick Barber

**Affiliations:** 1 Departamento de Ciencias Farmacéuticas, Facultad de Farmacia, Universidad de Valparaíso, Gran Bretaña 1093, Valparaíso, 2360102, Chile; 2 Department of Practice and Policy, UCL School of Pharmacy, Tavistock Square, London, WC1H 9JP, UK

## Abstract

**Background:**

There is a growing interest in high income countries to control expenditure on medicines by improving the rationale for their selection. However, in middle income countries with differing priorities and needs, little attention has been paid to this issue. In this paper we explore the policies and processes for the selection and use of medicines in a group of hospitals in Chile, a middle income country which has recently joined the OECD.

**Methods:**

A combination of qualitative and quantitative methods was used. A national survey questionnaire was distributed to investigate the role and operation of PTCs (Pharmacy and Therapeutics Committees). Interviews were conducted with key actors in the selection of medicines in large urban public hospitals.

**Results:**

The national survey had an overall response rate of 42% (83 out of 196), whilst 7 out of 14 hospitals participated in the qualitative study. High complexity hospitals are large urban hospitals; all of which claim to have a working PTC. The pharmacy offices are mainly involved in dispensing medicines with little involvement in clinical duties.

The interviews conducted suggest that the formulary of all the hospitals visited is no more than a stock list. PTCs are unable to influence the prescribing practices of doctors. Members do not feel prepared to challenge the opinions of specialists requesting a certain drug, and decisions are based primarily on costs. The inclusion of medicines in the clinical practice of hospitals is as a result of doctors bypassing the PTC and requesting the purchase of exceptional items, some of which are included in the formulary if they are widely used.

**Conclusions:**

There is an urgent need to develop medicine policies in hospitals in Chile. The procedures used to purchase medicines need to be revised. Central guidance for PTCs could help ensure a more rational use of medicines. PTCs need to be empowered to design formularies which cover all the clinical needs of doctors, training members in the analysis of scientific evidence beyond their own specialities. An influential PTC can take the appropriate measures and design workable policies to enforce a cost effective-use of resources.

## Background

Expenditure on medicines is one of the largest costs in healthcare. Consequently, countries worldwide are increasingly trying to control the use of resources spent on medicines. The World Health Organization (WHO) recommends the use of formulary systems to improve rationality in the use of drugs at regional, district and local levels [1]. These formulary systems should be designed and updated by multidisciplinary committees called Drug and Therapeutics Committees (DTC) or Pharmacy and Therapeutics Committees (PTC).

At the hospital level, the presence of these committees has been described in many high income countries, where DTCs are considered to improve the rationality of decisions [2-17]. Furthermore, the role of pharmacists and the presence of pharmacy services in hospitals have been associated with a more rational use of medicines [18,19].

Given the differing nature of health problems faced by rich and poor countries, there are key differences in the objectives of hospital DTCs when selecting medicines. On the one hand, decision-makers in low income countries need to focus on selecting medicines to save as many lives as possible, in which case the WHO list of essential medicines is extremely useful. On the other hand, committees of high income countries place emphasis not only on saving lives, but also on improving the quality of life for their chronically ill patients and promoting cost-effectiveness in prescribing, especially regarding the use of newer drugs not listed as essential by the WHO.

The objectives of hospital DTCs in middle income countries are probably somewhere between those of high and low income countries, with health needs requiring a migration from a live-saving essential pharmacotherapy, to one that not only saves lives, but also improves the quality of life of their populations. Thus the way that DTCs work in middle income countries could be directly related to the extent to which the population can access medicines that are not on the WHO’s essential list.

Such is the case in Chile, an upper middle income country according to the World Bank, which joined the Organization for Economic Cooperation and Development (OECD) in 2010. Membership of the OECD suggests that the country will engage in the process of developing policies to improve the quality of life of its population. Nevertheless, information about Chilean hospitals is virtually non-existent and therefore a study of decisions about medicines in Chile is a good opportunity to improve our understanding of how decisions are made in hospitals in a middle income country engaged in the process of migration mentioned above.

### The Chilean Health System

Since 1980, three health systems have coexisted in Chile; namely, a public system serving around 70% of the population, a private system serving approximately 27%, and the health system for the armed forces for around 3% of Chilean citizens [20]. The most important provider of secondary care is the public network of hospitals which covers most towns and cities in Chile; while large highly complex private hospitals are only found in the biggest cities. Public hospitals are classified by the Ministry of Health as high and low complexity hospitals. There are no official criteria for classifying hospitals as high or low complexity according to defined parameters. Instead, the classification seems to be based on how the network operates: low complexity hospitals aim to improve geographical coverage, while high complexity hospitals are regional or reference centres to which low complexity hospitals refer patients who require a more complex level of care.

### The health reform

A health reform was in progress at the time of the research, which aimed to ensure coverage for a list of catastrophic diseases by progressively implementing a programme called GES (meaning Explicit Guarantees in Health). In this programme, both private and public insurance systems are required to fully cover all costs in the treatment of a defined list of 69 diseases (at no cost to the patient), such as different types of cancer, HIV, diabetes and cystic fibrosis. The treatment of these diseases must be in accordance with clinical guidelines designed by a panel of experts.

### Pharmaceutical policies in Chile

For a medicine to be commercialized in Chile, the manufacturer needs to register their product with the National Institute for Health (ISP). The ISP is not a specialised medicines agency, but the agency in charge of overseeing the safety of every product that is consumed by humans and animals. At the time of the study, generic medicines were only required to present clinical studies performed for the original drug. Currently the ISP is implementing a National Agency for Medicines (ANAMED) and generic drugs will be required to prove bioequivalence in order to be granted market authorization in Chile.

Chile has a long tradition of pharmaceutical policies. In fact, the first National Formulary of Medicines was developed in Chile between 1965 and 1970, and later adopted by the WHO in 1977 [21]. Unfortunately, between 1973 and 2004 no pharmaceutical policies were in force, with the commercialization and use of medicines regulated almost exclusively by market laws. Currently, the National Formulary of Medicines is simply a list of medicines that have been granted market authorization in Chile and does not include any treatment guidelines for those drugs.

The National Medicines Policy published in 2004 (i.e. 4 years before the study) establishes that all hospitals, regardless of their complexity, must have a Pharmacy and Therapeutics Committee in charge of decisions about medicines. The PTC should be chaired by the medical director, the secretary of the committee should be the head of the Pharmacy Department, and all clinical department heads should be permanent members.

This study explores the policies and processes involved in the use of medicines in Chilean public hospitals and aims to explore the extent to which decisions concerning the formulary of medicines are rational, and how these decisions could be improved. Moreover, the study may also help decision makers to develop policies and procedures for the selection and use of medicines in other middle income countries.

### Research questions

1. What are the characteristics of Chilean Public hospitals in terms of the number of beds, the duties of the pharmacy office, the formulary systems, and the existence of multidisciplinary committees?

2. What are the central and local policies and procedures for the selection of medicines in Chilean public hospitals?

3. What are the formal and informal ways by which the use of a drug not previously used is commenced in Chilean public hospitals?

## Methods

Due to the virtual absence of published information about Chilean public hospitals, a combination of quantitative and qualitative methods was used to address these questions. A two-stage study was performed, comprising a survey followed by semi-structured interviews with purposively selected informants. By using these complementary methods it was possible to gather structured data concerning the aspects of Chilean hospitals that are described in the literature as factors that influence the innovativeness of institutions [8,10,22,23], and it was also possible to explore relevant issues and processes in context.

### Nationwide survey

#### Overview

A postal survey was designed in order to identify characteristics of Chilean Public hospitals. The questionnaire requested information on hospital size, complexity, and the existence of a PTC [8,10,22-28] (Additional file 1).

##### Sampling strategy and procedure

The survey was a population study, with the questionnaire sent to all 196 hospitals that appear on the Ministry of Health’s website.

##### Development of instruments

The first draft of the questionnaire was piloted with five hospital pharmacists, selected using a snowballing technique following initial contact with a hospital pharmacist previously known to a member of the research team. After corrections were made, the second draft was piloted and discussed with a further snowball sample of hospital pharmacists until no new issues emerged.

##### Data collection

In January 2008, the questionnaire with an explanatory letter from the research team, a letter from the Ministry of Health encouraging response, and pre-paid envelopes to return the questionnaire, were sent to all hospitals addressed to “The person in charge of the pharmacy office”. After two months, a second questionnaire was sent to non-respondents with a personalised letter encouraging response to the questionnaire.

##### Data processing and analysis

The responses to the questionnaire were entered and analysed using SPSS 18® software and descriptive procedures.

##### Ethics statement

The survey requested information about the hospital and did not include any questions which could involve patient data. The survey was then submitted to the Chilean Ministry of Health for ethics approval which was deemed unnecessary.

### Qualitative study

#### Overview

The results of the questionnaire suggest that high complexity hospitals are more likely than low complexity hospitals to innovate in their pharmacotherapy, and hence be more likely to deal with new medicines. Therefore, we conducted a qualitative study in high complexity hospitals in order to answer research questions 2 and 3. From May 2008 to August 2008, interviews were carried out with the chief of the Department of Pharmaceutical Policy at the Ministry of Health, members of PTCs, doctors who had applied for new medicines to be added to the formulary, nurses, and clinicians working in the hospital (Additional file 1).

##### Sampling strategy, procedure and recruitment

Different sampling strategies were used for the selection of cities, hospitals and interviewees inside hospitals. Firstly, for practical purposes only three cities could be selected for the study. The cities were: Santiago, the capital city located in the geographical centre of the country with approximately 6 million inhabitants; Valparaíso located on the coast 150 km from Santiago with a population of nearly one million; and Coquimbo, 450 km north of Santiago with less than half a million inhabitants. Thus, these urban hospitals represent different regions of the country where specialist doctors tend to be concentrated. Secondly, fourteen hospitals in these cities were selected using purposive sampling aiming to include at least one of the different types of high complexity hospitals present in those selected cities (i.e. child, cancer, psychiatric, and general hospitals). Thirdly, a snowballing strategy was employed to select hospital staff, starting with the chief pharmacists who then identified other members of the PTC. Doctors who had applied for a drug to be admitted to the formulary were identified from the minutes of PTC meetings. The last two doctors who had applied for a drug were contacted through the chief pharmacist.

Clinicians were identified by convenience sampling, in which the researcher approached the first doctor found after walking into the wards and asking them to participate in the project (all wards of the hospital were included). Interviews with doctors were continued until saturation of codes was reached (i.e. no new issues emerging), or in the cases of two hospitals (where recruiting doctors was difficult) until the planned visiting time for the hospital was over.

##### Development of instruments

Specific interview schedules were designed for pharmacists, members of the PTC, drug applicants, clinicians and nurses. The interview schedules included open questions about national and local policies regarding the use of medicines, formal and informal procedures for using medicines not included in the formulary, the purchase of medicines, and about how the pharmacy office oversees the use of medicines in the hospital.

##### Data collection

The interviews were carried out between April and July 2008 by a Chilean pharmacist and researcher with no previous working experience in hospital pharmacy in Chile, hence with no prejudgement about the possible answers to the interviews. The interviewer, trained in relevant research methods, maintained the principles of the qualitative enquiry while performing the interviews, limiting himself to encouraging interviewees to give more information about topics that emerged during interviews. Interviews were audio recorded with the consent of the interviewee; when permission was not granted, written notes were taken.

Minutes of PTC’s meetings held during the year 2007 were reviewed to identify medicines that had been included in the formulary during that year.

##### Data processing and analysis

Recorded interviews were transcribed verbatim and then analysed in their original language using the MAXQDA® qualitative analysis software. The analysis was carried out using a thematic approach identifying policies and procedures relating to the inclusion of drugs in the clinical practice of the hospitals. Subsequently, policies were sub classified into national and local policies, and procedures into formal and informal procedures. The analysis was carried out using an approach based on the Grounded Theory, coding the data into different hierarchies as suggested by the data itself. These hierarchies were the result of an iterative process in which accounts were classified into the different codes and then re-classified into sub-codes until no further issues emerged.

##### Ethics statement

The interview schedules did not contain any questions involving individual patient’s data. Nevertheless, we submitted the questions to the Ethics Committee of one of the participant hospitals; this committee established that no ethics approval was necessary for the study.

## Results

### Nationwide survey

#### Characteristics of samples and response rates

83 hospitals responded to our questionnaire, representing 48% of all high complexity hospitals and 40% of low complexity hospitals (42% overall). Geographically, the survey obtained responses from 14 out of the 15 administrative regions of the country, which represents a good picture of the different realities of public hospitals in Chile.

#### Chilean public hospitals

Respondent hospitals ranged from basic undifferentiated rural facilities with as few as six beds, to urban hospitals with a maximum of 720 beds. All the high complexity hospitals are located in urban areas (i.e. more than 10,000 inhabitants), and 54% of the low complexity hospitals are in urban areas. Table 1 summarizes the findings of the national survey regarding the characteristics of Chilean hospitals.

**Table 1:** Summary of characteristics of Chilean hospitals

	**High complexity hospitals**				**Low complexity hospitals**			
	N	Min	Max	Mean	N	Min	Max	Mean
Number of beds	28	116	720	340.6	51	6	317	63.8
Number of clinical units	27	4	25	10.5	53	0	9	3.6
Pharmacists per 100 beds	27	0.47	2.47	1.16	51	0	3.33	0.75
Expenditure per bed/year 2007 (US$)	26	1478	26891	6815	46	121	17928	2915

#### The hospital pharmacies

A pharmacist is in charge of the pharmacy office in all but one high complexity hospital. Conversely, only 48% of low complexity hospitals have a pharmacist in charge. On average, high complexity hospitals have 3.9 pharmacists (full-time equivalents) per hospital, while low complexity hospitals have 0.5. Moreover, the number of pharmacists per 100 beds is 1.16 for high complexity and 0.75 for low complexity hospitals.

The pharmacy offices are found to be concerned mainly with the dispensing of medicines and medical supplies, with the manufacturing of extemporaneous medicines, and the preparation of parenteral nutrition. Pharmacists are mostly involved in administrative work, although on their own initiative, some pharmacists are involved in clinical duties such as monitoring the use of antibiotics and therapies in critical care units.

#### Pharmacy and Therapeutics Committees

According to the survey, all the high complexity hospitals and 89% of the low complexity hospitals have a PTC. In terms of how long these committees have been established, 83% of high complexity hospitals have a committee actively working for 5 or more years, while only 39% of low complexity hospitals have a committee that has been working for five or more years. The professions of those working in the PTCs of hospitals that responded to the national survey are summarized in Table 2. Additionally, the frequency of meetings agreed by PTCs and the reported number of drug applications discussed in a typical PTC meeting are shown in Tables 3 and 4 respectively.

**Table 2:** Composition of PTCs in Chilean public hospitals

**Complexity level**	**Doctors**	**Pharmacists**	**Nurses/midwives**	**Dentists**	**Commercial/administrative professionals**	**Other professionals**	**No degree**
High	100% (28/28)	100% (28/28)	82% (23/28)	18% (5/28)	60% (17/28)	14% (4/28)	0% (0/28)
Low	100% (45/45)	56% (25/45)	84% (38/45)	44% (20/45)	33% (15/45)	22% (10/45)	53% (24/45)
Total	100% (73/73)	73% (53/73)	84% (61/73)	34% (25/73)	30% (22/73)	19% (14/73)	33% (24/73)

The figures represent the percentage of PTCs having one or more permanent members with the indicated profession.

**Table 3:** Agreed frequency of meetings of PTCs

	**High complexity hospitals**		**Low complexity hospitals**	
Frequency of agreed meetings	N	Percentage (valid responses)	N	Percentage (valid responses)
Bi-monthly	5	17.2	2	4.3
Monthly	12	41.4	8	17.0
Quarterly	10	34.5	22	46.8
Bi-annually	1	3.4	12	25.5
Other	1	3.4	3	6.4

**Table 4:** Reported number of drug applications discussed in a typical meeting of the PTC

	**High complexity hospitals**		**Low complexity hospitals**	
Number of drug applications discussed per meeting	Frequency	Percentage (valid responses)	Frequency	Percentage (valid responses)
No application discussed yet	0	0	5	10.6
One or less	10	35.7	14	29.8
Two or three	13	46.5	18	38.3
Four or five	3	10.7	6	12.8
More than five	2	7.1	4	8.5

## Drug expenditure

The annual expenditure on medicines in Chilean hospitals was estimated based on the responses to the survey and expressed as expenditure per bed. For high complexity hospitals the average expenditure per bed was US$6,815 during the year 2007, while for low complexity hospitals the average was US$2,914. The overall expenditure per bed was US$4,233.

### Qualitative study

#### Response rates

Only seven out of the 14 hospitals originally invited, agreed to participate in the study; namely, three regional hospitals, one cancer hospital, one children’s hospital and two general hospitals in the capital city. Altogether, the head of the Department of Pharmaceutical Policies at the Ministry of Health, seven pharmacists, nine members of PTCs, six applicants for drugs, five nurses and 41 clinicians were interviewed.

#### Medicines budget

The budget for medicines was calculated according to the historical expenditure plus an additional 6 to 7% of annual growth. Although some pharmacists reported that the budget has increased as a result of the GES programme, all of them agreed that the budget is insufficient as hospitals are facing an uncontrolled increase in debt.

#### Policies on the selection of medicines

##### Pharmacy and Therapeutics Committee

At the time of the study, hospitals were periodically audited to find out if they had a PTC by the Sub-secretariat of Health Network of the Ministry of Health. Nevertheless, no evaluation into how these committees actually function was undertaken at the time. Indeed, pharmacists and members of the committee seemed to only be aware that there is an obligation to have a PTC through those audits rather than through the Department of Pharmaceutical Policies.

All of the hospitals visited during the study have a working PTC that has been functioning for at least five years. These hospitals use a form stating the generic name, the number of potential patients, and the scientific evidence, as recommended by the national policy.

##### The formulary

Every hospital is required to have a list of the medicines that are used to treat patients, which translated literally into English would be called a “Therapeutic Arsenal”. If a medicine is on the list, the hospital is obliged by law to have a permanent stock of it and could be sued by a patient if a doctor prescribes the drug and it is not available in the hospital. However, according to pharmacists this information is not common knowledge and patients are not normally informed of this legal requirement. In addition to GES guidelines, only one of the seven participant hospitals had designed clinical guidelines for the treatment of their patients, but these guidelines were written by each clinical department and not by the PTC. Interviewees admitted that these guidelines are seldom used.

##### The GES programme

There is a different policy for medicines that are included in the GES programme. The clinical guidelines for each disease included in the programme recommend specific treatments to hospitals; however, the paying party (FONASA in the case of publicly insured patients) only reimburse medicines that are mentioned in the clinical guidelines, making the inclusion of these medicines in the formulary mandatory.

#### Formal Practices relating to the use of medicines

##### The Pharmacy and Therapeutics committee

The selection of medicines in the hospitals we visited is carried out, according to our interviewees, by a multidisciplinary committee referred to as the Pharmacy and Therapeutics Committee. Invariably, the chair of this committee is the hospital director and the secretary is the chief pharmacist. The heads of the various clinical departments are permanent members of the committee, with the exception of one hospital where the committee is formed by the chair, the secretary, a clinical pharmacologist and the head nurse.

“… ***we used to include all the heads of the clinical departments***, ***but they were on the committee only to ask for medicines***, ***they didn***’***t really contribute to the debate***…” (Chief pharmacist)

##### The application process

There was no consensus as to who is authorised to apply for medicines to be added to the formulary, some interviewees said that only doctors are authorised, while others said that any health professional can apply. This lack of consensus was generalised, with statements for both sides made in all hospitals. To make an application, applicants are required to fill in the form, submit it to the head of the clinical department for authorisation (signature) and submit it to the pharmacy office. In terms of the types of evidence that are usually submitted with the application form, the consensus is that applicants do not provide sufficient evidence to support the application.

“… ***well***, ***I can***’***t lie to you***, ***the evidence is normally weak***, ***very weak***…” (Member of the PTC)

Moreover, some pharmacists claimed that in certain cases doctors support their applications by writing the phrase “*because I need it*” on the form.

In two of the hospitals the committee’s secretary is responsible for providing the evidence to support the application. We asked applicants and pharmacists about the source of the information that is usually presented to the committee; the most common answer was “*scientific publications*” but without naming any specific publication. Only one applicant mentioned databases such as the Cochrane Collaboration and Web of Knowledge. Interestingly, all applicants and pharmacists mentioned the drug company’s representative as a source of scientific articles.

“… ***and representatives***, ***they always bring papers***, ***they are very useful***…” (Chief pharmacist)

##### The decision-making process

In all the visited hospitals, after the application is received by the committee’s secretary, the application is put on the next meeting’s agenda to be presented to the members. In the subsequent meeting, its inclusion on the TA is discussed, once members have had the chance to study the application. During the meeting where the application is discussed, the application is sometimes presented by the applicant and sometimes by the secretary of the committee. Following a discussion of the evidence submitted, a decision is reached by consensus. In one of the hospitals, interviewees mentioned the use of a formula to weigh the different aspects of the application such as cost, number of patients to be treated and the drug’s cost-effectiveness ratio.

When asked about the discussion preceding the decision, all the interviewees agreed that it is mainly centred on costs; in particular, members of the PTC pointed out that when the drug in question is out with their speciality, they do not feel they can contribute an authoritative opinion to the discussion, hence the focus on costs.

##### Perceptions of the PTC

The opinion of the PTC among members of the committee is very positive. They highlighted that the existence of the committee adds formality and transparency to the process of selecting medicines. However, the opinion of clinicians is less clear; when asked about the local policy to incorporate a drug into the TA, only 16 out of the 41 interviewed doctors were aware of a local policy involving a PTC. Out of the 16 doctors who were familiar with the policy, only seven could name the functions of the PTC. In addition, all of the doctors who were unaware of the policy thought that the policy for including a medicine in the TA was to “ask for it in the pharmacy”.

In addition, there was some criticism as to how the PTC works, with doctors mainly criticising the bureaucracy of the system; while pharmacists complained about the extra work load that the committee entails for them, as well as the slowness of the process.

##### The exceptional purchase procedure

In parallel to the formal application process for medicines not included in the TA, hospitals have a formal procedure through which doctors can request the purchase of a medicine not included in the TA. Doctors need to fill in a form requesting the drug and explaining their reasons for the request and submit the form to the pharmacy office. This request is then sent to the medical sub-director who decides whether or not the pharmacy office will buy it. According to doctors and pharmacists, the entire procedure takes just one day or less for the doctor to obtain the requested drug. All chief pharmacists agreed that this procedure is overused by doctors, and recognised that medical sub-directors rarely reject an application for exceptional purchases.

“… ***we had eight applications for drugs last year*** (***2007***)… ***For exceptional purchase***? ***Well more than 2***,***000***… ***way more***… ***I just can***’***t count them***” (Chief pharmacist)

Moreover, the number of requests for some medicines has grown steadily over time, overtly becoming part of the medicines normally used in the clinical practice of the hospital without approval by the committee, even though chief pharmacists are not keen to include them in the Therapeutic Arsenal because they create an economic burden for the hospital.

“… ***For me it***’***s also easier to buy medicines through the exceptional purchase***, ***they are normally more expensive drugs and if we include them in the TA everybody will start prescribing them***… ***the hospital can***’***t afford that***…” (Chief pharmacist)

It is important to point out that this is the result of the PTC including medicines in the formulary but without any restrictions on their use. Once a new medicine is on the TA, any doctor can prescribe it.

Moreover, due to the extra work load involved in processing many requests for a particular drug, in all the visited hospitals the committee decided to formally include the medicine in the formulary without a formal application for the drug, or by asking doctors to submit a formal application. In these cases the drug is included without a discussion of the evidence on the use of the drug. Some doctors and nurses raised the issue of the limited scope of medicines used in public hospitals, which in their opinion leaves them no alternative but to request the appropriate drug using the exceptional purchase protocol.

“… ***the Therapeutic Arsenal of public hospitals is really limited***, ***there are only basic drugs outdated long ago*** …” (H1.21 Clin 1)

#### Informal practices relating to the use of medicines

##### The private purchase of medicines

The most common informal practice mentioned by physicians in the hospitals in this study, is the external purchase of drugs. In this practice, the doctor issues a prescription for the patient or his/her relatives to buy one or more medicines in a community pharmacy, for use in the hospital. Even though this practice implies an agreement between doctor and patient, there was no mention of any informed consent.

It is not clear how common this practice is, since different kinds of interviewees had different opinions on the subject. On the one hand, 27 out of the 41 physicians interviewed in hospitals mentioned this practice as a common way for them to prescribe drugs not listed in the formulary. On the other hand, pharmacists and PTC members insisted it is rare.

**Figure 1:**
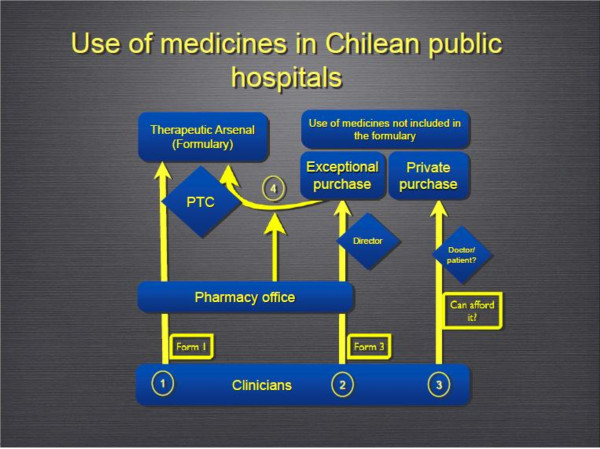
**Routes to include a drug in the clinical practice of Chilean public hospitals.**Three routes are available for doctors to prescribe a drug not included in the formulary of Chilean hospitals. (1) Doctors can apply to the PTC for a drug through the pharmacy office. (2) They can request the exceptional purchase of a medicine. (3) If they think the patient can afford the drug, they can ask them to buy the drug privately to be used in the hospital. The number of requests for exceptional purchases grew gradually over time for some drugs; to avoid the paperwork associated with processing too many requests, the pharmacy office asks the PTC for the inclusion of this drug into the formulary (4).

Nurses reported perceived frequencies of private purchases ranging from none to 20% of all prescriptions in their wards. Moreover, nurses pointed out that private purchases are usually for one of three reasons; namely, specific drugs not included in the TA, a temporary stock shortage of the drug in the hospital, and doctors simply wanting to prescribe drugs of “*better quality than those used by the hospital*”. Furthermore, in four cases, the nurses reported that the deciding factor as to whether or not patients receive private prescriptions, is if the doctor thinks the patient can afford to buy the drug or not (see Figure 1).

##### The use of free prescription samples

Similarly, the use of free samples to treat patients in hospitals was frequently mentioned in the interviews by clinicians and nurses, but pharmacists seem to be unaware of the use of free samples to treat patients. Doctors gather medical samples, probably from drug representatives visiting the hospital, and treat patients that cannot afford to pay for the medicines themselves with samples provided by pharmaceutical companies. Nevertheless, it was not possible to establish whether this is common practice from the interviews. In one of the visited hospitals, we frequently observed representatives of pharmaceutical companies in the hospital corridors giving doctors promotional plastic bags with unknown contents.

## Discussion

This study presents, for the first time, information on both the size and the complexity of Chilean hospitals and the control of medicines within them. The results of the qualitative study are, to the best of our knowledge, the first attempt to widen our knowledge on how decisions about medicines are made in public hospitals of a middle income country like Chile. Nevertheless, conducting research in a health system where people are not used to research was not without problems; a marked resistance of individuals to participate was observed during the fieldwork. Although the study does not identify the reasons for this resistance, we believe that including qualitative methods in the design is particularly appropriate to answer our research questions, instead of a questionnaire providing limited perspectives.

Despite this, we think that some biases could be present in the results of the qualitative study. Indeed, only seven out of the 14 hospitals agreed to participate in the qualitative study, with pharmacists of those hospitals expressing a high opinion of their PTCs. High complexity hospitals that responded to the questionnaire account for almost half the total number of those hospitals, which raises the question of whether the absence of a working PTC might be the reason for some hospitals not responding. Thus, there is a possibility that hospitals with less active PTCs could be underrepresented in this study.

### Policies

The national policy for the use of a PTC is clear and acknowledged by hospitals. Nevertheless, it seems that the policy is often viewed by hospitals as an obligation imposed on them as part of the administrative changes currently taking place in the public sector, rather than a useful tool to improve the rationality of decisions. In the local context, policies on the use of drugs are not present or not enforced in practice, with an absence of clinical guidelines to support doctors’ decisions to prescribe medicines included in the formulary. In fact, with the exception of expensive antibiotics, no prescribing restrictions were identified.

### The formulary

The formulary used in Chilean hospitals seems to be no more than a stock list. According to our results, the so called Therapeutic Arsenal does not include clinical guidelines to orientate doctors in the use of medicines within hospitals. Moreover, the obligation to keep a permanent stock of the medicines that appear in the TA might be discouraging hospitals from updating their TAs, but this could be overcome by including medicines along with appropriate policies for their use. In addition, it is also possible that Therapeutics Arsenals do not cover the whole spectrum of medicines needed to treat patients in those hospitals, obliging doctors to seek medicines outside the hospital.

Even when the reasons for prescribing medicines that are not included in the Therapeutic Arsenal are unclear, the lack of obligation to prove the bioequivalence of generic medicines in Chile may be relevant. With hospitals using mostly generic drugs, the distrust doctors show towards these types of drugs is an important issue that can partially explain why doctors prescribe drugs not included in the TA of public hospitals.

Additionally, PTCs in Chile need to develop formulary systems which can effectively cover all the pharmacotherapy needed in hospitals, avoiding the exceptional purchase of medicines. Moreover, it is necessary to improve our understanding of doctors’ reasons for prescribing medicines that are not included in the Therapeutic Arsenal; taking the appropriate measures to avoid this practice will result in a more rational use of medicines.

**Figure 2:**
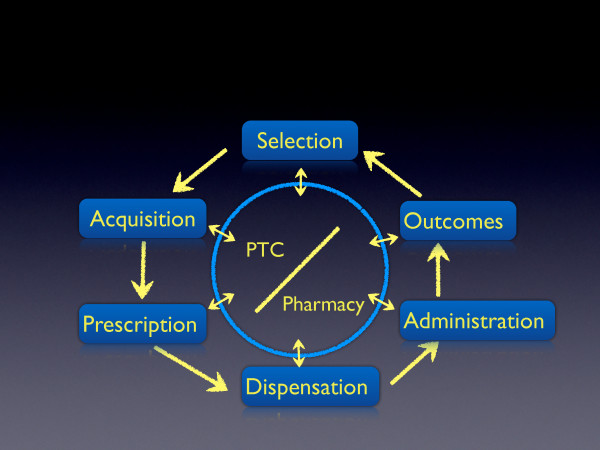
**Information**-**flow diagram.** Instead of a linear process, the use of medicines in Chilean hospitals should be continuous where information on each stage of the use of medicines is shared by the PTC, the pharmacy office and clinical services, providing continuous feedback about the use of medicines included in the Therapeutic Arsenal.

Moreover, it is important to improve the communication between the PTC, clinicians and the pharmacy office. At the time of the study, the use of medicines appeared to be a linear process in which medicines were selected, prescribed, dispensed, and then administered; but there was no feedback about the outcomes achieved with newly included medicines. Thus, to achieve a more rational use, there should be continuous communication between the PTC, the Pharmacy office and clinicians about the use of drugs (see Figure 2).

### The Pharmacy and Therapeutics Committee

Jenkings and Barber describe the lack of skills of DTC members to analyse and contextualise scientific information [29]. Coincidentally, members of the studied PTCs self-reported that the evidence discussed in PTC sessions is often difficult to apply to the Chilean reality, and they do not feel able to challenge the opinions of specialists in areas other than their own. This is clearly a barrier to making more rational decisions in Chilean committees, highlighting the need to train members in the appraisal and analysis of scientific information beyond the scope of their specialities.

There is a positive perception among members of the PTC about having a committee in charge of decisions to include a drug in the TA. However, it seems that for the group of hospitals under study, the PTC does not have enough authority to influence prescribing in their own hospitals, leaving innovation in the use of drugs (i.e. using drugs not previously used in the hospital) to the criteria of individual doctors or groups of doctors. This lack of authority could be the result of poor argumentation about reasons to include or not a medicine in the formulary and the lack of communication about the decisions of health professionals working in the hospital, undermining the legitimacy of the committee’s decisions.

This need for legitimacy can be improved with an ethical approach called the ‘Accountability for Reasonableness [30]; this approach was put forward to give greater legitimacy to health technology decisions. This approach advises that decisions must be effectively communicated to doctors, stating the rationale for the decision and giving the opportunity to appeal. By using an ethical approach such as the Accountability for Reasonableness, decisions made by the PTC can gain greater legitimacy, gradually building the authority needed to influence prescribing in the group of hospitals included in this study.

Moreover, several authors suggest different strategies to improve the decision-making process for including a drug in the formulary. In this respect, Tan et al. suggest that institutions should clearly state a hierarchy of priorities before any discussion on including a drug is made [31], and Schiff et al., propose a Formulary Drug Application Tool, which they suggest might help DTC members to evaluate claims about drugs made during the application process, giving a framework to improve the level of the discussion [32].

### The GES programme

Medicines included in the GES programme are normally drugs not included in the TA of the hospitals under study; in practice, they are imposed on hospitals, contradicting what the National Policy of Medicines of 2004 states regarding the selection of medicines. Nevertheless, since decisions to include a drug into the TA are normally based almost exclusively on costs, it is highly probable that hospitals would not include those drugs in their TAs if they were not obliged to. Thus, the obligation of hospitals to include GES drugs could be improving the access to more innovative medicines in those hospitals.

## Conclusions

The national policy for selecting medicines in public hospitals is, as recommended by the WHO, through local multidisciplinary committees; however, there is a need to improve the ability of committees to influence prescribing towards evidence based practice. More central guidance on the selection of new medicines could help committees to make more rational decisions, and to design policies and protocols for the use of medicines based on the best available evidence.

There is also a need to revise the mechanisms for purchasing medicines in public hospitals to improve their quality and to circumvent abuses of the exceptional purchasing system. In general, the lack of regulation in the Chilean market for medicines may be having a negative impact on the access to good quality medicines for the majority of Chilean citizens.

More research is needed to better understand how medicines are used in Chilean hospitals; for instance, it is fundamental to establish doctors’ reasons for prescribing medicines that are not included in the formulary. Only by understanding how medicines are used in Chilean hospitals, can the appropriate policies be put in place to improve rationality in the use of medicines.

The results of this study can serve as a basis for further research into how middle income countries can change their priorities, means and needs in terms of healthcare and the way medicines are used. Such research can be used to develop appropriate policies for an optimal use of medicines and to improve health outcomes.

## Competing interests

The authors declare no competing interest.

## Authors’ contributions

All of the interviews and analysis were carried out by JFC. FS collaborated with methodological guidance; NB with methodological guidance and the analysis of interviews. All authors read and approved the final manuscript.

## Pre-publication history

The pre-publication history for this paper can be accessed here:

http://www.biomedcentral.com/1472-6963/13/10/prepub

## Supplementary Material

Additional file 1 **Appendix.** The appendix shows the English translation of the questionnaire used in the nationwide survey and the interview schedules used during the qualitative study. Click here for file
