# Supplementary material for: Selection of medicines in Chilean public hospitals: an exploratory study
Source: BMC Health Serv Res. 2013 Jan 7;13:10. doi: 10.1186/1472-6963-13-10 (PMC3542206; doi:10.1186/1472-6963-13-10)
Supplement: Additional file 1 — Appendix. The appendix shows the English translation of the questionnaire used in the nationwide survey and the interview schedules used during the qualitative study. [file 1472-6963-13-10-S1.docx]

**Questionnaire used in the nationwide survey**


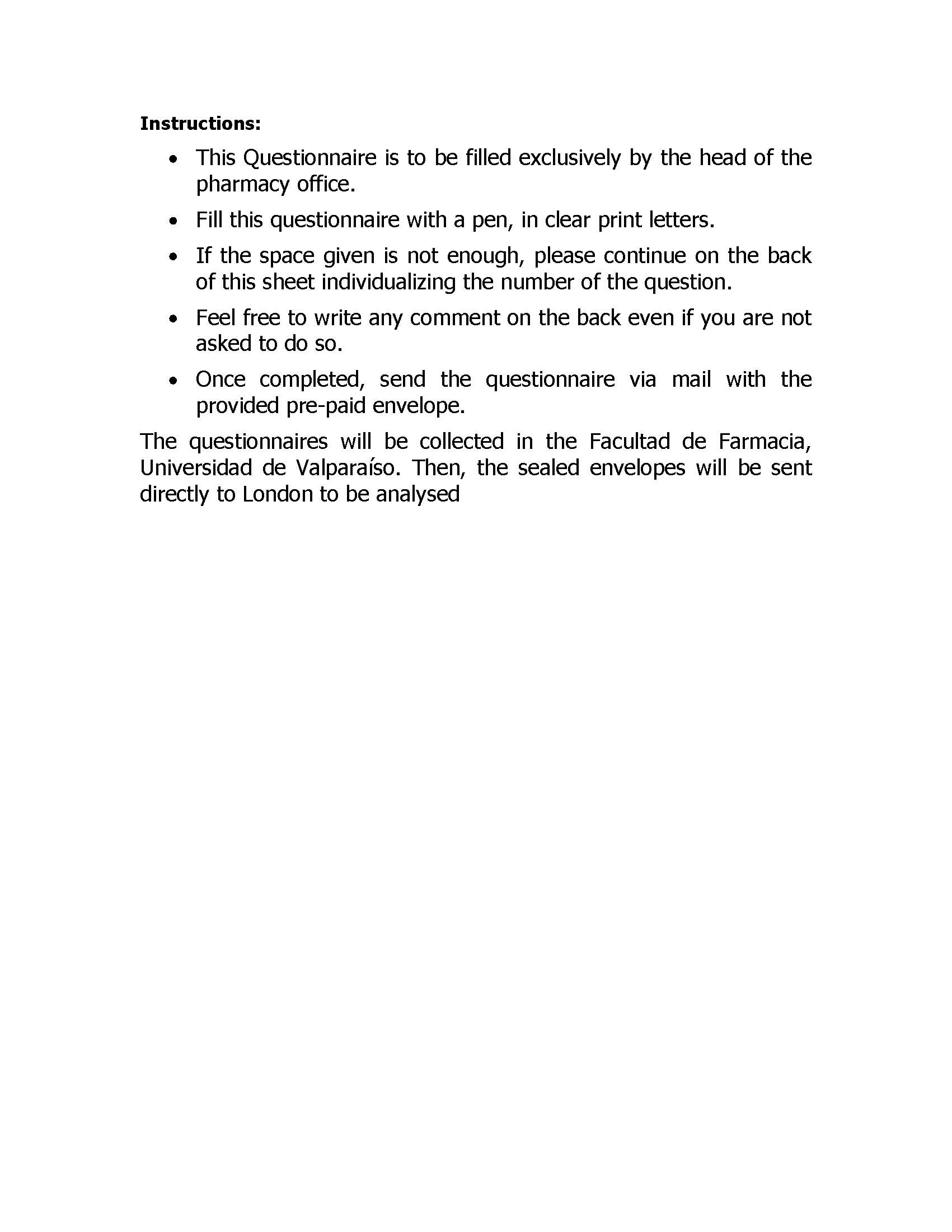

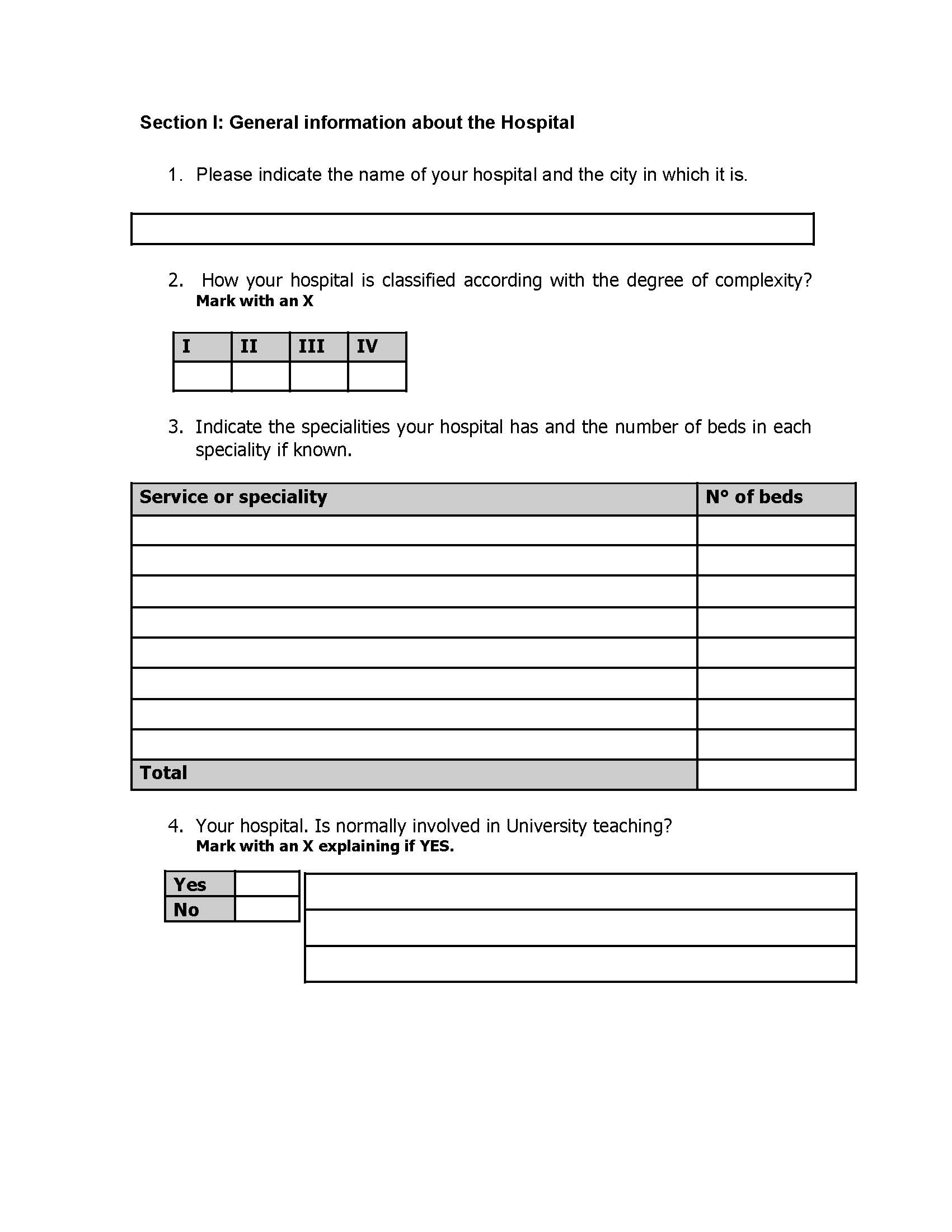


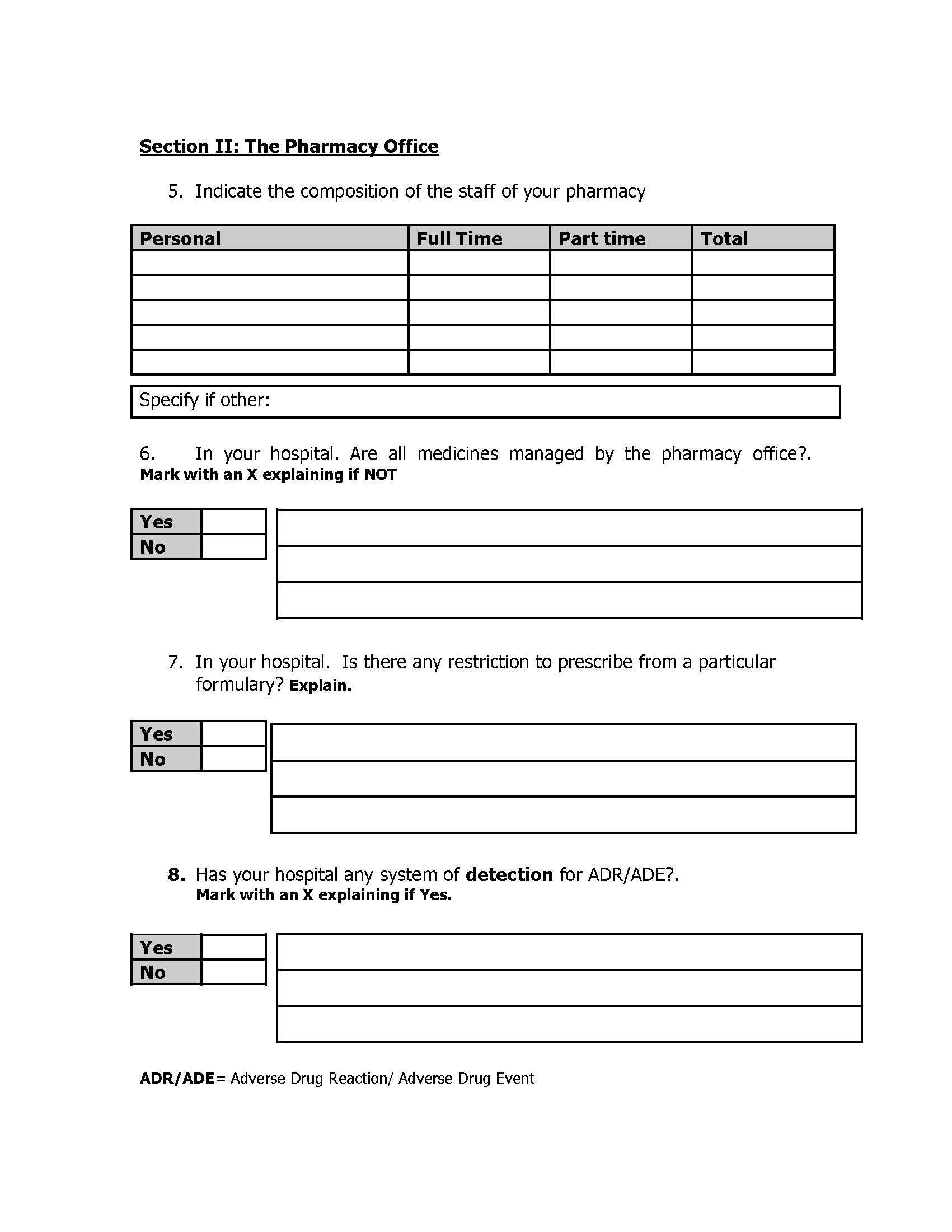

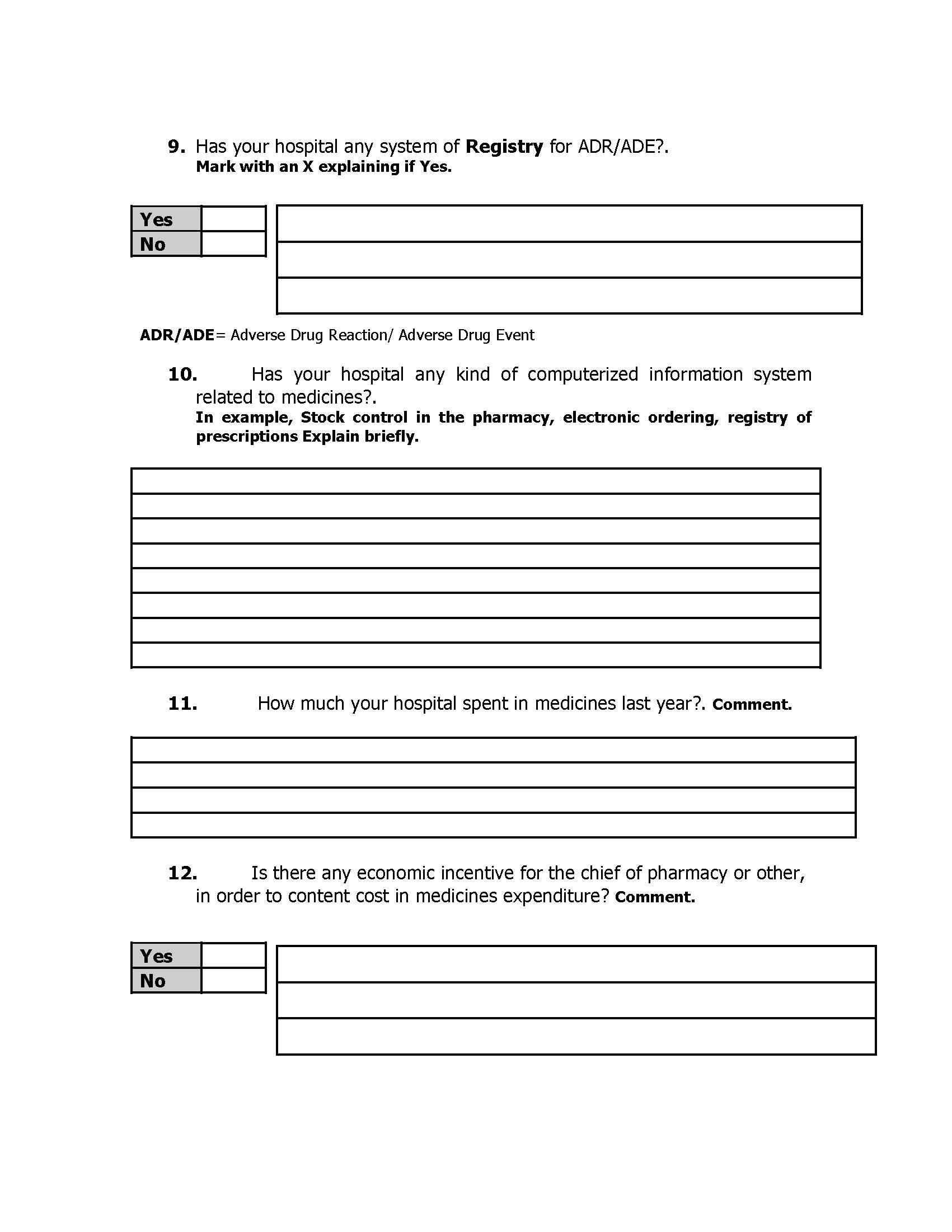


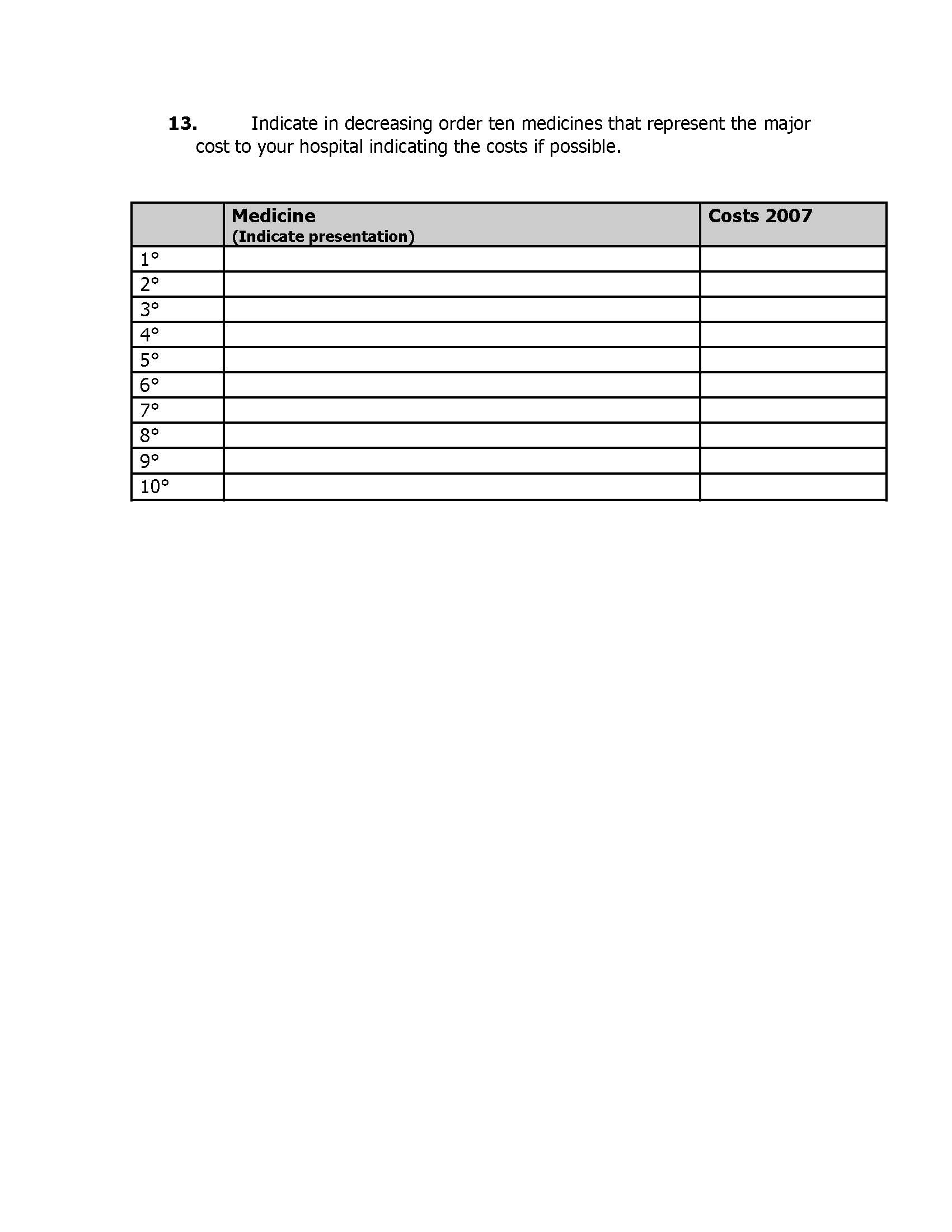

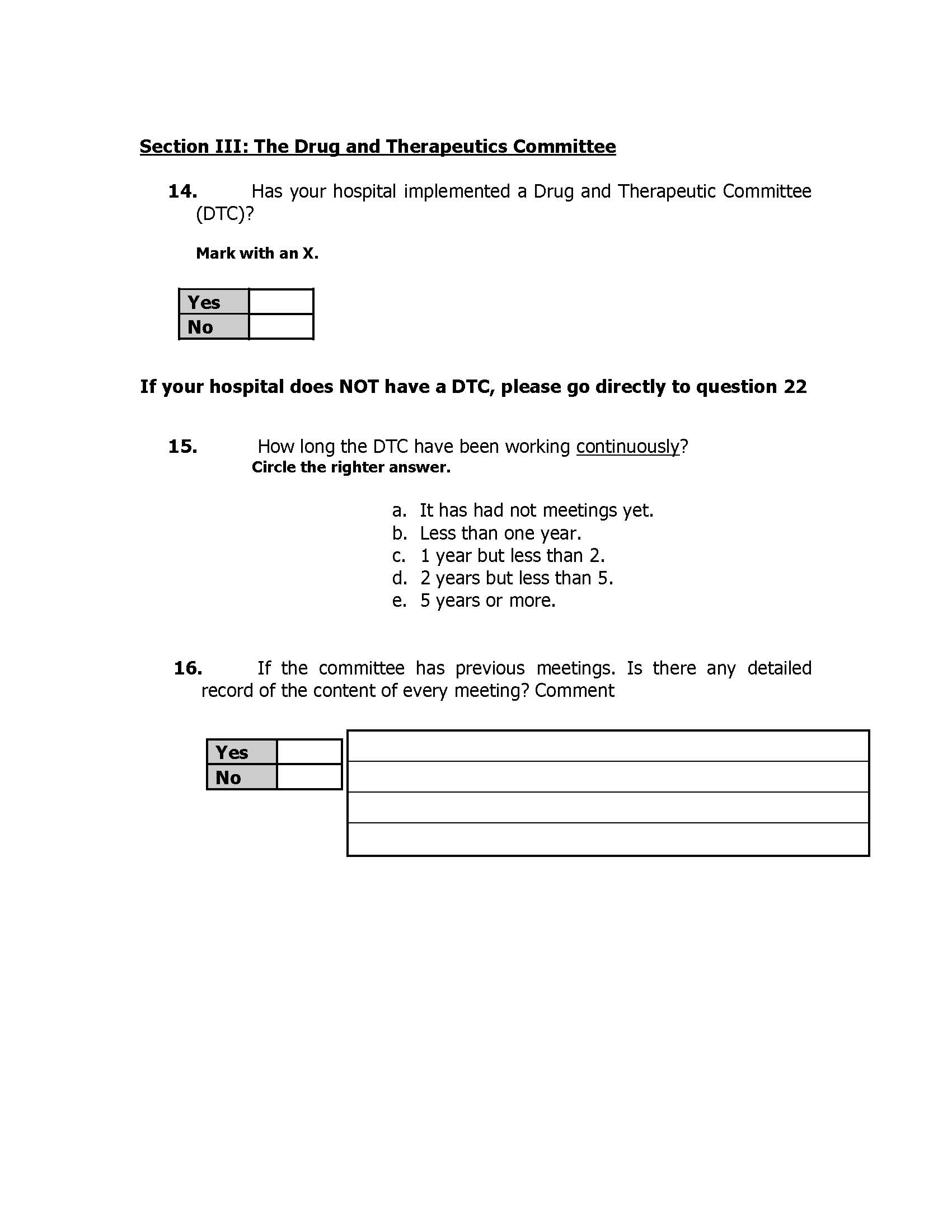


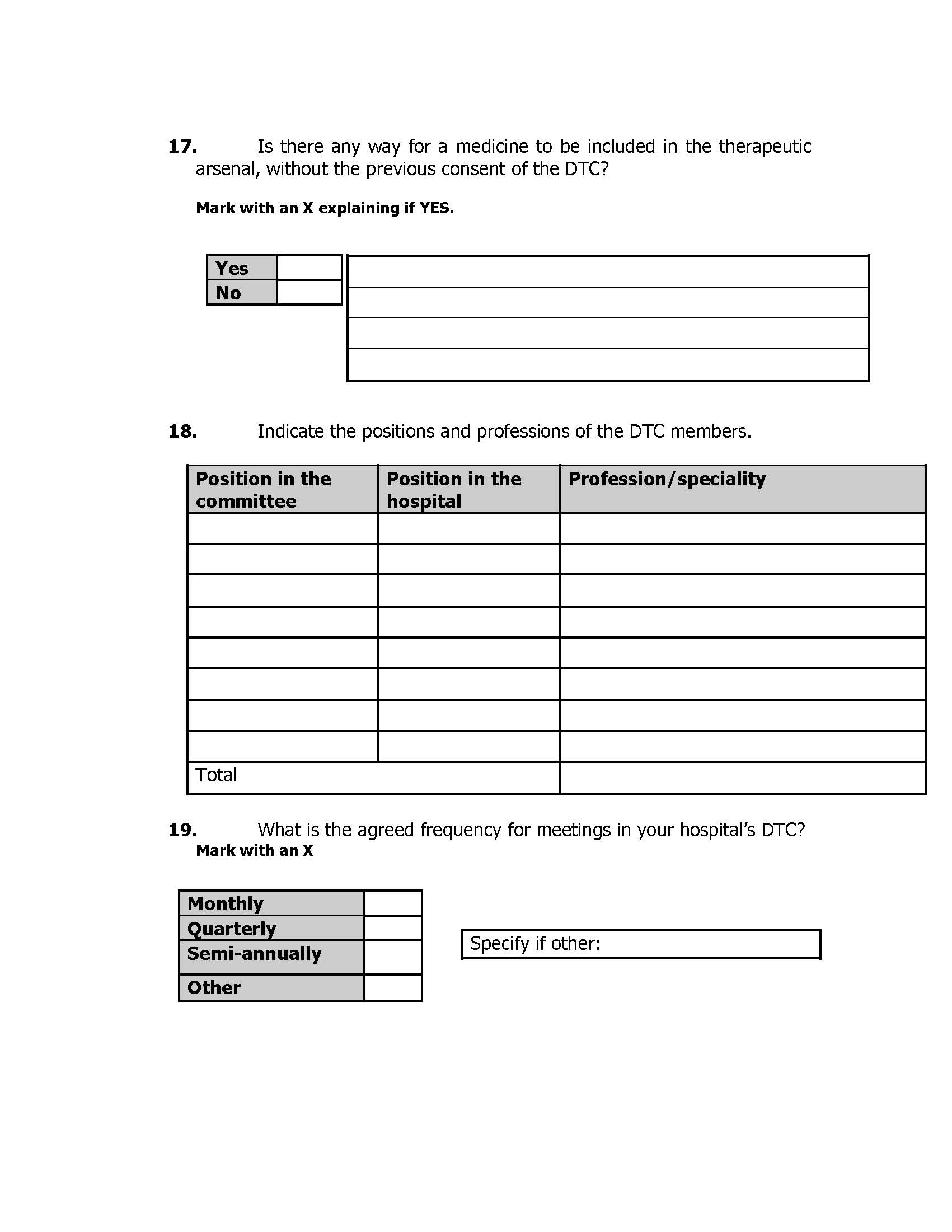

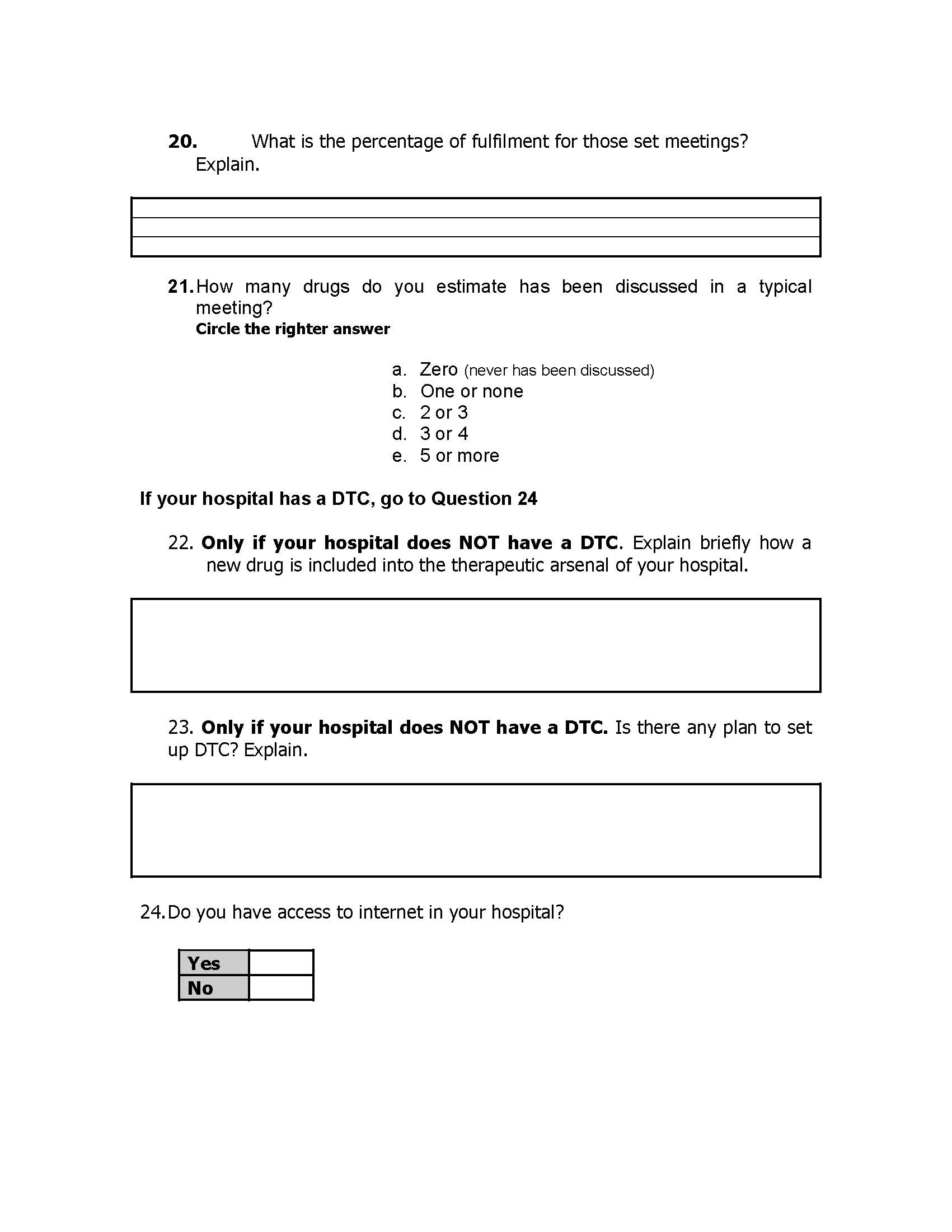


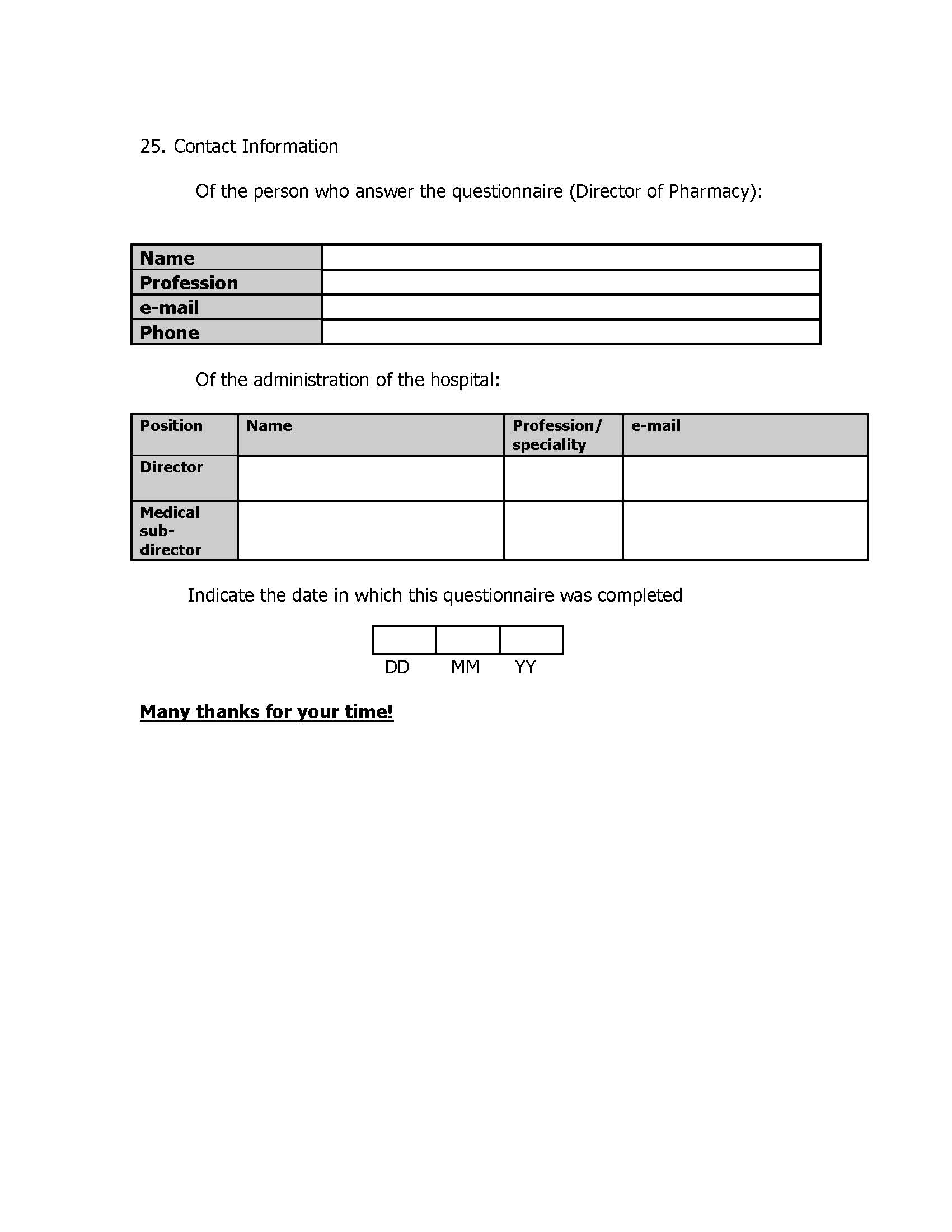


Interview Schedules used during the fieldwork of the Qualitative Research

| **Interview with the Chief pharmacist**   1. **Policies regarding incorporation of new drugs.**    1. Can you describe current national and/or regional policies regarding adoption of new drugs?    2. Can you comment positive aspects of these policies?    3. Can you comment negative aspects of these policies?    4. Can you describe local policies for the adoption of a new drug?    5. Can you comment positive aspects of these policies?    6. Can you comment negative aspects of these policies?    7. What is the policy of hospital regarding visits of drug representatives? Comment.    8. Do you think this policy is fulfilled in the practice?    9. Do these policies affect in the practice decisions about new drugs? How? 2. **Decision-making about new drugs.**    1. When deciding to incorporate a medicine in the arsenal. Is there a special treatment for decisions regarding new drugs?    2. Where decisions are taken to incorporate or reject a new drug for you hospital?    3. Are there any exceptions to this rule?    4. What about drugs used for AUGE plan?    5. Is there any way in which a new medicine not previously approved by the DTC may be prescribed to an in-patient? In example, if the patient buy the drug outside the hospital.    6. What is the policy of the hospital in that regard?    7. Would you describe the process by which a new drug is proposed to the DTC?    8. Who is entitled to do the application?    9. Who is not entitled?    10. Is there any form for the application?    11. Does the applicant need to attach any antecedent to the application?    12. Who are members of the DTC in your hospital?    13. Would you describe the process inside the DTC to decide about a new drug that has been proposed?    14. Are there any exceptions to this rule? Explain.    15. Is there any written statement about the roles of members of the DTC?    16. What is your role inside the DTC?    17. What is the information that the DTC take into account when deciding about a new drug? Explain.    18. Who is the most influent member of the DTC when deciding about a new drug? Explain 3. **Purchases of drugs**    1. What was the budget/expenditure for 2007?    2. Would you please describe how the drug budget is defined in your hospital?    3. What are the mechanisms the hospital has to purchase medicines?    4. What are the methods of payment the hospital have?    5. Would you please mention main providers the hospital has?    6. Does the hospital have any preference for some of these providers? Why?    7. How is the stock of medicines defined?    8. Is there any financial incentive for members of the DTC for cost-containment? 4. **The Pharmacy office.**    1. How many people work in the pharmacy office?    2. Can you name their professions and positions?    3. What are the normal duties of the pharmacy office?    4. Does the pharmacy office keep any statistic of drug use? Explain.    5. To what extent this statistics include all medicines used inside the hospital?    6. Does the pharmacy office make any kind of following up for drugs? Explain.    7. May you mention computational systems the pharmacy office have and its functions.    8. Does the hospital have any kind of system for pharmacovigilance? Explain.    9. Is there any procedure by which a drug may be removed from the therapeutic arsenal? Explain.    10. What would be reasons to remove a drug from the therapeutic arsenal? |
| --- |

| **Interviews with members of the DTC**   1. **Policies Regarding incorporation of new drugs**    1. Can you please describe current national and/or regional policies regarding adoption of new drugs?    2. Can you comment positive aspects of these policies?    3. Can you comment negative aspects of these policies?    4. Can you describe local policies for the adoption of a new drug?    5. Can you comment positive aspects of these policies?    6. Can you comment negative aspects of these policies?    7. What is the policy of the hospital regarding visits of drug representatives?    8. Do you think this policy is fulfilled in the practice? 2. Do you think these policies affect in the practice decisions about new drugs? How? 3. **Decision-making about new drugs.**    1. What is your position in the DTC?    2. What is your role in the DTC?    3. Would you describe the process inside the DTC to decide about a new drug that has been proposed?    4. Are there any exceptions to this rule? Explain.    5. What is the information that the DTC take into account when deciding about a new drug? Explain.    6. How the final decision about new drugs is reached in the DTC?    7. Who is the most influent member of the DTC when deciding about a new drug? Explain.    8. Is there any way in which a new medicine not previously approved by the DTC may be prescribed to an in-patient? In example, if the patient buy the drug outside the hospital.    9. What is the policy of the hospital in that regard?    10. Would you describe the process by which a new drug is proposed to the DTC?    11. Who is entitled to do the application?    12. Who is not entitled?    13. Is there any form for the application?    14. Does the applicant need to attach any antecedent to the application?    15. Would you please describe how the drug budget is defined in your hospital?    16. What are the mechanisms the hospital has to purchase medicines?    17. What are the methods of payment the hospital have?    18. Would you please mention providers the hospital has?    19. Does the hospital have any special agreement with some of those providers?    20. Does the hospital have any preference for some of these providers? Why?    21. Is there any financial incentive for members of the DTC for cost-containment?    22. Is there any procedure by which a drug may be removed from the therapeutic arsenal? Explain.    23. What would be reasons to remove a drug from the therapeutic arsenal? 4. **General information about the member of DTC**    1. What is your profession and academic degree?    2. Do you have any postgraduate studies? Explain.    3. Do you attend scientific or professional conferences regularly? Can you mention recent conferences you have attended? |
| --- |

| **Interviews with physicians**   1. **Applicants for new drugs** 2. **Application process**    1. Would you describe the process by which a new drug is proposed to the DTC?    2. Is there any form for the application?    3. Do you need to attach any antecedent to the application?    4. How did you first hear about the last drug you apply for at the DTC?    5. Were did you get information about the mentioned drug that make you decide to apply for?    6. As a decision-maker. Can you mention the positive and the negative aspects of the DTC?    7. As a policy-maker. Can you mention the positive and the negative aspects of the DTC? 3. **Clinicians** 4. **General matters**    1. What do you think about local policies regarding incorporation of new drugs?    2. What can you do if you think a new drug must be in the therapeutic arsenal?    3. Can you apply the drug to the DTC?    4. Is there any way by which you can prescribe a new drug that it is not already in the therapeutic arsenal? Explain.    5. As a decision-maker. Can you mention the positive and the negative aspects of the DTC?    6. As a policy-maker. Can you mention the positive and the negative aspects of the DTC? |
| --- |

| **Interview with the head of the Department of Pharmaceutical Policies in the Health Ministry of Chile**   1. **About the department of pharmaceutical policies and medical professions.**    1. What are the goals of the Department?    2. Can you tell me about how policies are set?    3. What kind of information is used to set these policies?    4. Would you tell me about how decisions about new drugs are taken in Chilean Hospitals?    5. What are the current policies about incorporation of new drugs in hospitals?    6. Do you have any feedback mechanism to know about how these policies work in the practice? |
| --- |

Figure 4: Interview Schedule for Complementary Interviews

| **Second Interview with the Chief Pharmacist**   1. What can you tell me about the number of Forms #1 and Forms #3 that are normally received in the pharmacy office? 2. How often do you think that requests for Exceptional Purchase are approved by the director?    - What are your impressions about this procedure? 3. Is there any therapeutic class that is more commonly requested through Exceptional Purchase? 4. When a drug that has been constantly requested through exceptional purchase, do you include it in the formulary?    - How often does this happen?    - Is there any particular therapeutic class that is more often included this way? 5. Is there any list of drugs in your hospital that have restrictions to be prescribed? 6. Who is in charge of the supply of medicines in your hospital?  - What is his/her profession?  1. Who is in charge of setting the technical requirements for bids placed in Chilecompras?  - What technical requirement(s) do you have to ensure quality of medicines?   **Interview with the Chief Nurse**   1. Are there medicines bought privately in your department?    - What do you think are the reasons for buying medicines outside the hospital?    - How many patients are today being treated with medicines bought privately?    - Is there a particular therapeutic class that is more commonly purchased privately? 2. What are your perceptions about buying medicines privately? |
| --- |
